# Loss of the Nuclear Envelope Protein LAP1B Disrupts the Myogenic Differentiation of Patient-Derived Fibroblasts

**DOI:** 10.3390/ijms232113615

**Published:** 2022-11-06

**Authors:** Gülsüm Kayman Kürekçi, Aybar C. Acar, Pervin R. Dinçer

**Affiliations:** 1 Department of Medical Biology, Hacettepe University Faculty of Medicine, Ankara 06100, Turkey; 2 Centre Hospitalier Universitaire Sainte-Justine Research Center, Department of Molecular Biology, University of Montreal, Montreal, QC H2X 3E4, Canada; 3 Cancer Systems Biology Laboratory, Graduate School of Informatics, Middle East Technical University, Ankara 06531, Turkey

**Keywords:** LAP1, *TOR1AIP1*, nuclear envelope, myogenic differentiation, muscular dystrophy

## Abstract

Lamina-associated polypeptide 1 (LAP1) is a ubiquitously expressed inner nuclear membrane protein encoded by *TOR1AIP1*, and presents as two isoforms in humans, LAP1B and LAP1C. While loss of both isoforms results in a multisystemic progeroid-like syndrome, specific loss of LAP1B causes muscular dystrophy and cardiomyopathy, suggesting that LAP1B has a critical role in striated muscle. To gain more insight into the molecular pathophysiology underlying muscular dystrophy caused by LAP1B, we established a patient-derived fibroblast line that was transdifferentiated into myogenic cells using inducible MyoD expression. Compared to the controls, we observed strongly reduced myogenic differentiation and fusion potentials. Similar defects were observed in the C2C12 murine myoblasts carrying loss-of-function LAP1A/B mutations. Using RNA sequencing, we found that, despite MyoD overexpression and efficient cell cycle exit, transcriptional reprogramming of the LAP1B-deficient cells into the myogenic lineage is impaired with delayed activation of *MYOG* and muscle-specific genes. Gene set enrichment analyses suggested dysregulations of protein metabolism, extracellular matrix, and chromosome organization. Finally, we found that the LAP1B-deficient cells exhibit nuclear deformations, such as an increased number of micronuclei and altered morphometric parameters. This study uncovers the phenotypic and transcriptomic changes occurring during myoconversion of patient-derived LAP1B-deficient fibroblasts and provides a useful resource to gain insights into the mechanisms implicated in LAP1B-associated nuclear envelopathies.

## 1. Introduction

The nuclear envelope (NE) is composed of a double membrane, including an inner nuclear membrane (INM) with its inner surface lined by nuclear lamina and an outer nuclear membrane (ONM). Despite the wide and ubiquitous expression of the NE in a variety of tissues, mutations in the components of the NE cause diverse tissue-specific pathologies, including muscular dystrophies, cardiomyopathies, neuropathies, and lipodystrophies [1,2]. Studies suggest that this tissue specificity likely results from the altered interaction of NE proteins with tissue-specific protein partners or chromatin domains [1,3]. Moreover, disruption of tissue-specific epigenetic regulation has been shown in laminopathies [4].

Lamina-associated polypeptide 1 (LAP1) encoded by *TOR1AIP1* (torsinA-interacting protein 1) is a type II transmembrane protein of the INM that was initially identified in rat as three isoforms (LAP1A, LAP1B, and LAP1C) [5]. The nucleoplasmic domain of LAP1 interacts with the nuclear lamina, while its lumenal domain enables the formation of heterocomplexes with torsinA chaperones and stimulates their ATPase activity [6,7,8,9,10]. In contrast to rodents, human *TOR1AIP1* encodes only two isoforms: LAP1B (long isoform) and LAP1C (short isoform). As a product of an alternative downstream transcription start site, LAP1C has a truncated nucleoplasmic domain compared to LAP1B [11,12]. During mitosis, LAP1 isoforms are localized in the mitotic spindle and centrosomes [10], and both knockdown and overexpression of LAP1B/C lead to post-mitotic abnormalities in vitro [13,14,15]. In response to genotoxic stress, LAP1B/C interacts with telomeric repeat-binding factor 2 and co-localizes with DNA damage markers in micronuclei and nuclear blebs [16].

The first mutation in *TOR1AIP1* was identified in a family affected by progressive muscular dystrophy, joint contractures, and cardiomyopathy, which leads to the complete absence of LAP1B while expression of LAP1C is preserved [17]. Additional mutations specifically disrupting LAP1B were rarely reported in patients with muscular dystrophy, cardiomyopathy, and congenital myasthenic syndrome [18,19,20]. Moreover, a missense mutation putatively affecting the LAP1–torsinA interaction was identified in a patient with dystonia and cardiomyopathy [21]. The spectrum of LAP1-associated disorders was broadened with the identification of biallelic mutations affecting both LAP1 isoforms in individuals presenting with an early onset multisystemic, progeroid-like syndrome that includes neurological impairment, heart defects, hearing loss, cataract, and skin manifestations [22,23]. Hence, clinical presentation seems to correlate with the specific isoforms affected, as the loss of both LAP1 isoforms leads to a severe syndromic phenotype while specific loss of LAP1B mainly affects striated muscle.

In mice, constitutive knockout of *Tor1aip1* is perinatally lethal while conditional early embryonic knockout of *Tor1aip1* in skeletal muscle results in postnatal skeletal muscle growth defects [24,25,26]. Despite being ubiquitously expressed, LAP1 isoforms are detected at different ratios according to the differentiation level of the tissues or cells examined. While LAP1C expression is higher compared to LAP1B in undifferentiated cancer cell lines, such as SH-SY5Y, HeLa, and SKMEL-28, LAP1B is the predominant isoform in heart and brain, and its expression increases during murine neuronal and skeletal muscle differentiations (17,18). In addition, LAP1 has been shown to interact with the INM protein EMERIN responsible for Emery–Dreifuss muscular dystrophy (EDMD) [25]. Altogether, these findings strongly suggest a tissue selective role of LAP1B in muscle. However, previous studies did not focus on the specific absence of LAP1B, and in vitro models enabling the investigation of myogenic defects in patient-derived LAP1B-deficient cells are currently not available.

To address the specific roles of LAP1B during myogenic differentiation, we established an in vitro model of LAP1B-associated muscular dystrophy by converting patient-derived fibroblasts expressing only LAP1C into myogenic cells through MyoD overexpression. We showed that, although the LAP1B-deficient cells effectively exit the cell cycle upon MyoD induction, they present major fusion and differentiation defects compared to the controls. Impaired myogenic differentiation in the mutant cells is accompanied by the formation of micronuclei and, ultimately, cellular mortality. Time-course transcriptomic profiling revealed delayed and inefficient activation of the major myogenic differentiation regulator *MYOG,* as well as other muscle-specific genes. Pathway analysis showed dysregulations of protein homeostasis, extracellular matrix organization, and chromosome organization in the mutant cells.

## 2. Results

### 2.1. Myogenic Differentiation and Fusion Defects in LAP1B-Deficient Cells

Direct reprogramming of differentiated cells by overexpression of myogenic regulatory factors (MRFs; i.e., MyoD, Myf5, myogenin, and MRF4) recapitulates the steps of myogenesis [27]. Myogenesis involves the commitment of proliferating cells to myogenic differentiation, and the withdrawal of proliferating cells from the cell cycle is followed by cell fusion to ultimately form multinucleated myotubes [27,28]. Overexpression of MyoD in non-muscle cells, such as fibroblasts and liver, fat and nerve cells, is sufficient for their conversion into functional myoblasts and myotubes [29,30,31]. MyoD-mediated myoconversion of patients’ fibroblasts is especially useful as a resource for modeling muscular dystrophies and assessing therapeutic approaches [32,33,34,35].

**Figure 1:**
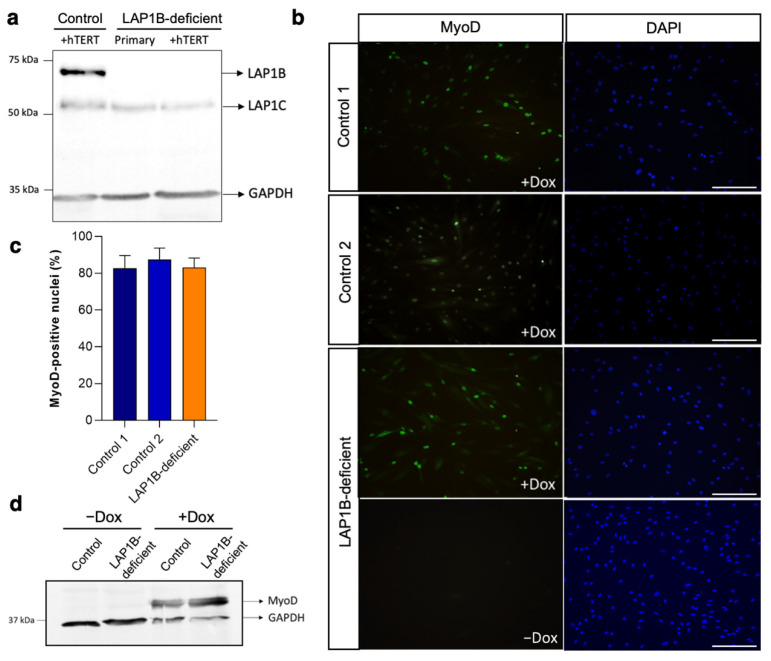
Immortalization and myoconversion of the control and patient-derived LAP1B-deficient fibroblasts. (**a**) Protein expression of LAP1 isoforms in the control immortalized (+hTERT) and patient-derived primary and immortalized (+hTERT) fibroblasts, as shown after Western blotting; GAPDH was used as a loading control. (**b**) Immunofluorescence staining of MyoD in the control and LAP1B-deficient fibroblasts at 18 h after induction with doxycycline (+Dox) supplementation in a differentiation medium; the nuclei are stained with DAPI. No MyoD induction was detected in the cells maintained in the differentiation medium without doxycycline supplementation (−Dox). Scale bar: 20 μm. (**c**) Percentage of MyoD-positive nuclei in the control and LAP1B-deficient cells after 18 h of doxycycline-mediated MyoD induction. (**d**) Protein expression of MyoD in the control and LAP1B-deficient cells before (−Dox) and 48 h after (+Dox) doxycycline supplementation in the differentiation medium, as shown after Western blotting. GAPDH was used as a loading control.

To establish a human in vitro model of LAP1B-associated muscular dystrophy, we immortalized primary fibroblast cells derived from two healthy individuals and patient-derived fibroblasts carrying a biallelic LAP1B-specific loss-of-function mutation (c.186delG) in *TOR1AIP1* [17] (Figure 1a), and transduced them with a doxycycline-inducible *MYOD1* for myoconversion [32]. Similar transduction efficiencies (>83%) were achieved between the control and LAP1B-deficient cells, as shown by the quantification of MyoD-positive nuclei after doxycycline supplementation (Figure 1b,c). No illegitimate MyoD expression was detected in the fibroblasts incubated in a differentiation medium without doxycycline supplementation (Figure 1b,d).

**Figure 2:**
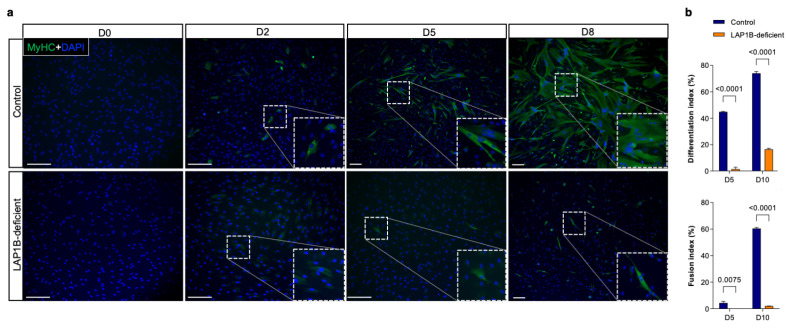
Myogenic differentiation of the control and patient-derived LAP1B-deficient fibroblasts. (**a**) Immunofluorescence staining of MyHC in the control and LAP1B-deficient cells at day 0 (D0), day 2 (D2), day 5 (D5), and day 8 (D8) after MyoD induction. The nuclei are stained with DAPI. The insets show MyHC-positive cells. Scale bar: 20 μm. (**b**) Fusion index (percentage of nuclei in MyHC-positive cells with at least two nuclei relative to the total number of nuclei) and differentiation index (percentage of nuclei in MyHC-positive cells relative to the total number of nuclei) in the control and LAP1B-deficient cells at day 5 (D5) and day 10 (D10) of differentiation. *n* = 3, where n represents the number of independent experiments, and *p*-values were determined using a two-way ANOVA followed by Bonferroni post hoc tests.

First, the myogenic differentiation potential of the control and mutant cells was monitored using the expression of myosin heavy chains (MyHC) as a myogenic differentiation marker (Figure 2a). As expected, no MyHC expression was detected in the uninduced control and mutant fibroblasts at day 0. In the controls, cells expressing MyHC were first detected two days after MyoD induction, indicating a commitment to myogenic lineage. At day 5, formation of multinucleated cells expressing MyHC in the controls indicated myogenic fusion. Finally, at day 8, the control cells formed mature multinucleated myotubes with differentiation and fusion indices of 76% and 60%, respectively (Figure 2a,b). In contrast to the controls, MyHC expression was rarely detected in the LAP1B-deficient cells until day 5 of MyoD induction (Figure 2a). At day 8, MyHC expression was restricted to 16% of the mutant cells, with only 2% of these containing more than one nuclei, indicating a major fusion defect (Figure 2a,b). Indeed, both differentiation and fusion indices at day 5 and day 10 were strongly reduced in the mutant cells compared to the controls (Figure 2b). To test whether myogenic differentiation was impaired or only delayed in the LAP1B-deficient cells compared to the controls, the mutant cells were further cultured in a differentiation medium with continuous MyoD induction for 17 days (Figure S1). Starting from day 10, cellular mortality was observed and, at day 17, more than 80% of cells had died, with only 28% of surviving cells expressing MyHC (Figure S1). These results showed that the control fibroblasts were efficiently transdifferentiated into mature myotubes within eight days, whereas myoconversion of the LAP1B-deficient fibroblasts was notably disrupted, and these cells failed to complete differentiation and, ultimately, died after prolonged exposure to the differentiation cues.

### 2.2. Impaired Differentiation of Tor1aip1-Mutant C2C12 Myoblasts

**Figure 3:**
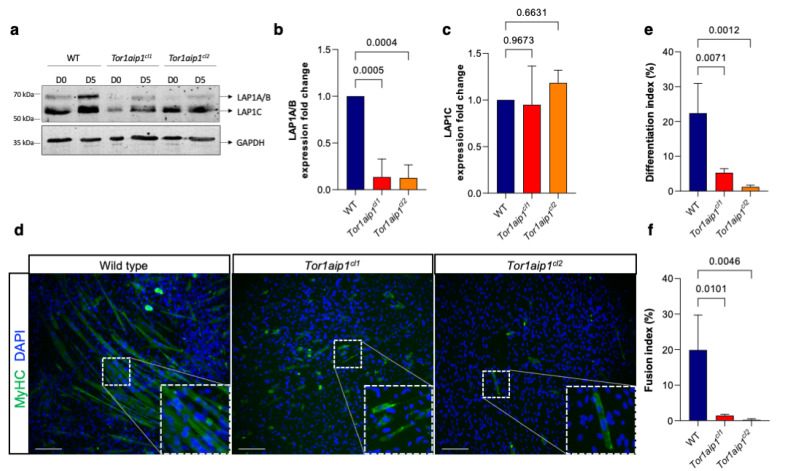
Generation and differentiation of *Tor1aip1*-mutant C2C12 myoblasts. (**a**) Protein expression of the LAP1 isoforms in two clones bearing different *Tor1aip1* loss-of-function mutations (*Tor1aip1^cl1^* and *Tor1aip1^cl2^*) at day 0 and day 5 of differentiation, as shown after Western blotting; GAPDH was used as a loading control. (**b**,**c**) Bar graphics show expression fold changes of (**b**) LAP1A/B and (**c**) LAP1C normalized to GAPDH in the mutant clones relative to the wild type. *n* = 3, and *p*-values were determined using a one-way ANOVA followed by Tukey’s post hoc tests. (**d**) Immunofluorescence staining of MyHC in the wild-type and *Tor1aip1*-mutant C2C12 myoblasts at day 5 of differentiation. The nuclei are stained with DAPI. The insets show MyHC-positive cells. Scale bar: 20 μm. (**e**) Differentiation index (percentage of nuclei in MyHC-positive cells relative to the total number of nuclei) and (**f**) fusion index (percentage of nuclei in MyHC-positive cells with at least two nuclei relative to the total number of nuclei) in the wild type and *Tor1aip1*-mutant C2C12 myoblast clones at day 5 of differentiation. *n* = 3, and *p*-values were determined using a one-way ANOVA followed by Tukey’s post hoc tests.

To study the effect of the loss of LAP1B in myoblast cells, two mutant C2C12 murine myoblast lines were created using CRISPR-Cas9 genome editing. In mice, LAP1A and LAP1B are expressed from a common transcription start site, and LAP1C is transcribed from a downstream alternative transcription start site [6,12]. Two mutant C2C12 myoblast clones harboring loss-of-function mutations in the first coding exon for LAP1A/B in *Tor1aip1* (*Tor1aip1^cl1^* and *Tor1aip1^cl2^*) were created (Figure S2). Significant downregulation of LAP1A/B was achieved in the *Tor1aip1^cl1^* and *Tor1aip1^cl2^* compared to the wild type (7.7-fold and 8-fold decrease, respectively), with no significant difference in LAP1C expression (Figure 3a–c). As previously reported in other studies, we found that LAP1C was the predominantly expressed isoform in the proliferating wild-type myoblasts and, upon induction of differentiation, LAP1A/B expression increased, supporting their critical role during the differentiation process (Figure 3a) [24]. Myogenic differentiation and fusion potentials of the wild-type and *Tor1aip1*-mutant myoblasts were monitored for five days through MyHC expression (Figure 3d). Both differentiation and fusion indices significantly decreased in the mutant clones compared to the wild-type cells (Figure 3e,f). In conclusion, loss of LAP1A/B alone was sufficient to impair myogenic differentiation and fusion in both myoconverted human fibroblasts and C2C12 murine myoblasts.

### 2.3. Overview of the Transcriptome Profile of Transdifferentiating Fibroblasts

**Figure 4:**
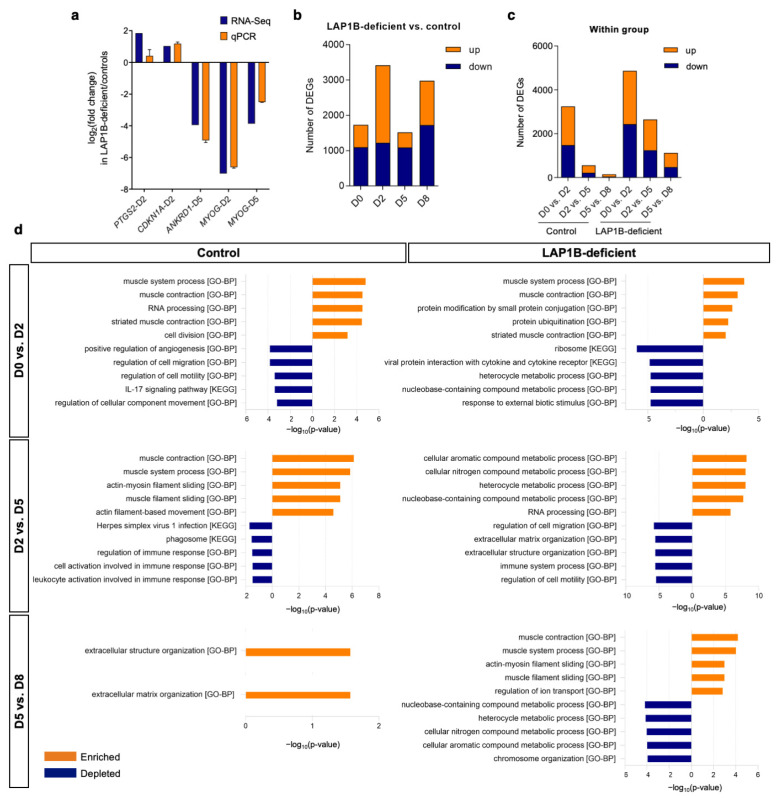
Time-course transcriptome profile of the control and LAP1B-deficient cells throughout MyoD-induced differentiation. (**a**) Validation of RNA-seq expression fold changes using qPCR. Log_2_ values of expression fold changes obtained in the RNA-Seq and qPCR of *PTGS2* at day 2, *CDKN1A* at day 2, *ANKRD1* at day 5, and *MYOG* at day 2 and day 5 in the LAP1B-deficient cells relative to the controls. Expression was normalized to *GAPDH* (*n* = 3). (**b**) Number of DEGs in the LAP1B-deficient cells compared to the controls at each day of differentiation. (**c**) Number of DEGs between consecutive days of differentiation within the controls and within the LAP1B-deficient cells. (**d**) Top five most significantly enriched and depleted GO-BP or KEGG categories at consecutive days of differentiation in the controls and LAP1B-deficient cells, which were obtained from gene set enrichment analyses using the Kolmogorov–Smirnov test and GeneTrail 3.2.

To identify putative genes and pathways underlying the defective myogenic differentiation observed in the myoconverted LAP1B-deficient cells, we reconstituted the time-course transcriptome profile of the control and mutant cells throughout their differentiation. We determined four time points for RNA sequencing representing the key stages of myogenic differentiation: day 0 for proliferating fibroblasts; day 2 for commitment to myogenic lineage; day 5 for myogenic fusion; and day 8 for terminal maturation of myotubes. Genes were considered to be significantly differentially expressed when the q-value was <0.05 and the linear expression fold change was ≥2. RNA sequencing results were validated for the selected genes at the selected time points using RT-qPCR (Figure 4a). First, we examined differentially expressed genes (DEGs) in the LAP1B-deficient cells compared to the control cells at each static time point (Figure 4b, Table S2). In the LAP1B-deficient proliferating fibroblasts at day 0, 1731 genes were differentially expressed compared to the controls; of these, 1094 were downregulated and 637 were upregulated. At day 2, 3419 genes were differentially expressed in the LAP1B-deficient cells compared to the controls, with 1224 downregulated and 2195 upregulated genes. At day 5, 1519 genes were differentially expressed in the mutant cells compared to the controls, with 1087 downregulated and 432 upregulated genes. Finally, at day 8, 2979 genes were differentially expressed in the mutant cells compared to the controls, with 1726 of them being downregulated and 1253 being upregulated (Figure 4b).

To gain more insight into the intrinsic differences that might influence the course of myogenic differentiation in each cell line, DEGs between consecutive time points were separately determined within the controls and the LAP1B-deficient cells (Figure 4c, Table S3). In the control cells, 3246 genes were differentially expressed between day 0 and day 2, corresponding to a withdrawal from the cell cycle and myogenic commitment. During the transition between day 2 and day 5 corresponding to the myogenic fusion stage, 562 genes were differentially expressed. Only 153 DEGs were found between day 5 and day 8 when myotube maturation took place. As expected, the most extensive transcriptional change occurred during the first transition when cells were committed to differentiation. During subsequent transitions (day 2 vs. day 5, and day 5 vs. day 8), the number of DEGs gradually decreased, reflecting the progressive completion of the differentiation program at the transcriptomic level. A similar pattern was observed in the LAP1B-deficient cells, except that the number of DEGs at each transition was higher compared to the controls (4867, 2650, and 1128 DEGs for day 0 vs. day 2, day 2 vs. day 5, and day 5 vs. day 8, respectively) (Figure 4c).

Overall, a substantial number of genes were differentially expressed in the LAP1B-deficient cells compared to the controls throughout differentiation. At the static time points, the number of downregulated genes in the mutant cells was higher than the number of upregulated genes for all of the days compared, suggesting impaired transcriptional activation. Furthermore, the number of DEGs at each stage of differentiation decreased with time in both groups, indicating an initial boost of transcriptional reprogramming that became gradually attenuated as terminal differentiation was reached. The higher number of DEGs in the LAP1B-deficient cells compared to the controls at each transition might reflect an attempt to transcriptionally compensate for the myogenic differentiation defects despite the continuous MyoD overexpression.

### 2.4. Transcriptome Profiling Reflects Incomplete Reprogramming of LAP1B-Deficient Cells

To identify which critical alterations in the gene expression program might cause the myogenic reprogramming defects in the mutant cells, we analyzed the temporal expression pattern of genes during differentiation within each group separately, and then compared the DEGs and their associated functions (Figure 4c, Table S3). We determined the top five most significantly enriched and depleted GO-Biological Process (Gene Ontology) and KEGG (Kyoto Encyclopedia of Genes and Genomes) categories in the control and mutant cells during each stage of differentiation (day 0 vs. day 2, day 2 vs. day 5, and day 5 vs. day 8) (Figure 4d).

In the control cells, upon induction of differentiation from day 0 to day 2, DEGs were enriched for muscle system processing and contraction (e.g., *KCHN2, MYOD1, MYOG, DES, TNNT3,* and *MYH8*), RNA processing (e.g., *ESRP2, CELF4,* and *APOBEC2*), and cell division (e.g., *CCND2, CDK1, SYCE2,* and *TOP2A*), and were depleted for cell migration (e.g., *IL6, CEMIP,* and *CDH5*) (Figure 4d). This indicates a commitment of the control cells to myogenic differentiation and fusion. Subsequently, between days 2 and 5, DEGs were essentially enriched for muscle contraction and filament sliding (e.g., *MYL1, MYH7, MYH2, TNNI1,* and *MYL3*), indicating synthesis of the components of the contractile machinery and maturation of cells, and were depleted for regulation of immune response (e.g., *HLA-DPA1, PIANP,* and *CD74*). Finally, during terminal differentiation from day 5 to day 8, the only significantly enriched category was extracellular matrix organization (e.g., *ASPN, CILP, EDIL3, POSTN,* and *COL16A1*), and no significantly depleted category was found (Figure 4d). These results indicated that, upon MyoD induction, the control fibroblasts were efficiently committed to myogenic differentiation, as reflected by the enrichment of genes involved in muscle contraction in the initial stages. Activation of muscle-specific genes was accompanied by downregulation of cell motility and immune response, and followed by modulation of the extracellular matrix in the terminal maturation stage.

In the LAP1B-deficient cells, an initial upregulation of genes involved in muscle contraction (e.g., *KCNH2, SCN4A, TNNT1, MYL4,* and *TTN*) was unexpectedly accompanied by increased protein ubiquitination (e.g., *TRIM36, FBXL16, RNF165, FBXL22,* and *UBE2QL1*), and decreased ribosomal activity (e.g., *RPL10, RPS2,* and *RPL38*) and heterocyclic compound metabolic activity (e.g., *IDO1, TTPA,* and *NNMT*) (Figure 4d). At the second stage of differentiation, the initial activation of myogenic genes was interfered with increased cellular aromatic compound metabolic process (e.g., *DUOX2* and *NMRK2*) and RNA processing (e.g., *AICDA, APOBEC2,* and *FAM207A*). At the same stage, downregulations of cell migration (e.g., *NRG1, LRRC15,* and *CEACAM1*), extracellular matrix organization (e.g., *MMP3, FBLN2,* and *LUM*), and immune response (e.g., *MUC20, CD160,* and *TLR1*) were observed (Figure 4d). From day 5 to day 8, despite impaired myogenic fusion and differentiation at the phenotypic level in the mutant cells, an upregulation of some muscle contractile genes (e.g., *TTN, CACNA1S, MYH8, LMOD2,* and *MYL1*) was detected, along with decreased nucleobase metabolism (e.g., *UBE2T, MND1,* and *M1AP*) and chromosome organization (e.g., *TOP2A, KIFC1, NUSAP1, KIF18B,* and *BUB1B*) (Figure 4d). In comparison to the transcriptome profile of the controls, these results indicated that protein and RNA metabolisms were affected in the early stages of differentiation in the LAP1B-deficient cells, followed by a disruption of extracellular matrix and chromosome organizations in the latter stage.

**Figure 5:**
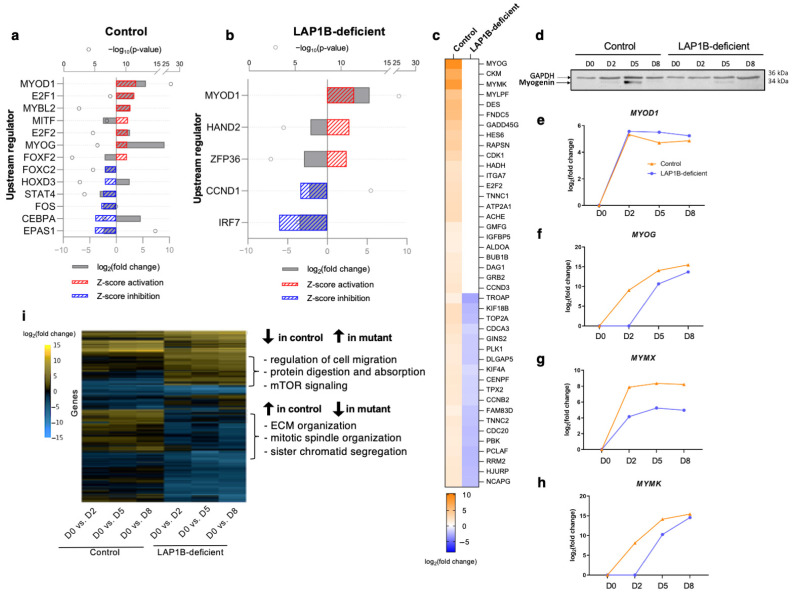
Upstream regulator and time-course regression analyses. (**a**,**b**) Upstream transcription regulators were predicted to be affected in the (**a**) control and (**b**) LAP1B-deficient cells during the transition from day 0 to day 2 of differentiation using Ingenuity Pathway Analysis. The upper X-axis shows −log_10_ of *p*-values (circles) for each upstream regulator. The lower X-axis shows log_2_ of expression fold changes (grey bars) and predicted transcriptional activation Z-scores (dashed red bars show activation, i.e., z-score > 2; dashed blue bars show inhibition, i.e., z-score < −2). (**c**) Heat map showing expression fold changes of DEGs in the MyoD network during the day 0–day 2 transition in the control and LAP1B-deficient cells. Genes that are upregulated in the controls, and downregulated or unchanged in the LAP1B-deficient cells are displayed. (**d**) Protein expression of myogenin in the control and LAP1B-deficient cells at days 0, 2, 5, and 8 of differentiation; GAPDH was used as a loading control. (**e**–**h**) RNA expression fold changes of (**e**) *MYOD1*, (**f**) *MYOG*, (**g**) *MYMX,* and (**h**) *MYMK* throughout differentiation as determined using RNA-Seq. (**i**) Clustered heat map showing expression fold changes of DEGs identified using time-course regression analysis. The top three enriched GO and KEGG categories for DEGs with opposite expression trends in the control and LAP1B-deficient cells throughout differentiation are shown.

As a master regulator of myogenic differentiation, MyoD drives myogenesis in cooperation with other MRFs by interacting with chromatin remodeling factors at muscle-specific loci to modulate chromatin accessibility [36]. As some muscle-specific genes seemed to be activated in the LAP1B-deficient cells despite the impaired differentiation, we aimed to analyze in more detail candidate genes that could be relevant to the observed phenotype. We performed an upstream regulator analysis to identify the master transcription regulators and predict whether they were activated or inhibited according to the gene expression profile [37]. We identified significantly activated (Z-score > 2) or inhibited (Z-score < −2) upstream transcription regulators during the first stage of differentiation in the controls and the LAP1B-deficient cells (Figure 5a,b). In the control cells, *MYOD1* was the most significantly activated upstream regulator, followed by *E2F1, MYBL2, MITF, E2F2, MYOG,* and *FOXF2*, while the significantly inhibited upstream regulators were *FOXC2, HOXD3, STAT4, FOS, CEBPA,* and *EPAS1* (Figure 5a). In the LAP1B-deficient cells, *MYOD1* was also the most significantly activated upstream regulator, followed by *HAND2* and *ZFP36*, while *CCND1* and *IRF7* were the significantly inhibited upstream regulators (Figure 5b). This showed that, in the LAP1B-deficient cells, *MYOG* (myogenin) transcriptional network failed to activate despite successful *MYOD1* activation. Myogenin is one of the four MRFs, and MyoD and myogenin can reciprocally activate each other’s expression, while cooperatively acting on the activation of overlapping genes [38,39]. To find which MyoD downstream genes were affected in the LAP1B-deficient cells, we analyzed DEGs within the MyoD network. Among 153 genes identified in the MyoD network, 42 genes were upregulated in the controls but either downregulated or unchanged in the mutant cells (Figure 5c). Among the DEGs, *MYOG* was the most highly upregulated gene of the MyoD network in the controls, followed by *MYMK* (myomaker), *CKM* (muscle creatine kinase), *DES* (desmin), and *FNDC5* (fibronectin type III domain containing 5); none of these were activated in the LAP1B-deficient cells at day 2. Downregulation of *MYOG* in the LAP1B-deficient cells compared to the controls was confirmed at the mRNA and protein levels (Figure 4a and Figure 5d). *MYMK* (or *TMEM8C*, myomaker) and *MYMX* (myomixer) are the main muscle fusogenic genes bound by both MyoD and myogenin, but activation of *MYMK* is mainly dependent on myogenin [40,41]. Consistently, transcriptional activation of *MYOG* and *MYMK* were correlated with each other throughout differentiation in both the control and mutant cells, while *MYMX* (myomixer) was not affected by the reduced expression of *MYOG* in the LAP1B-deficient cells (Figure 5e,f). Altogether, these results showed that *MYOD1* was efficiently activated in the LAP1B-deficient cells upon overexpression; however, delayed and insufficient activation of *MYOG* and downstream muscle-specific genes, such as *MYMK*, *CKM,* and *DES*, might contribute to their differentiation and fusion defects.

Lastly, we aimed to specifically uncover gene clusters with divergent expression trends between the control and LAP1B-deficient cells by performing a time-course regression analysis (Table S4). A total of 863 genes were identified in the regression analysis, and a clustered heat map revealed gene clusters displaying opposite expression trends between the control and mutant cells during differentiation (Figure 5i). Throughout differentiation, 548 genes had a positive expression trend (i.e., were mainly upregulated) in the control cells, but remained unchanged or were downregulated in the LAP1B-deficient cells (Figure 5i). These genes were associated with extracellular matrix organization (e.g., *COL1A1, DAG1,* and *LAMB1*), mitotic spindle organization (e.g., *BUB1B, AURKB, CENPA,* and *CENPU*), and sister chromatid segregation (e.g., *TOP2A, ESPL1,* and *NUSAP1*). In contrast, the expression of 315 genes was either downregulated or remained constant in the control cells, while being upregulated in the LAP1B-deficient cells throughout differentiation (Figure 5i). These genes were associated with regulation of cell migration (e.g., *LRRC15, CEMIP, FAM89B,* and *SEMA6D*), protein digestion and absorption (e.g., *SLC36A1, COL13A1, PGA3,* and *PGA4*), and mTOR signaling (e.g., *PRR5, EIF4EBP1, SGK1,* and *RNF152*). Taken together, the transcriptome analyses suggested an impairment of a temporally coordinated myogenic differentiation process in the LAP1B-deficient fibroblasts. In addition, the mutant cells displayed a disrupted protein homeostasis with increased ubiquitination and decreased protein translation upon MyoD induction; this was accompanied by a potentially compensatory increase in mTOR signaling.

### 2.5. LAP1B-Deficient Fibroblasts Efficiently Withdraw from the Cell Cycle upon MyoD Expression

**Figure 6:**
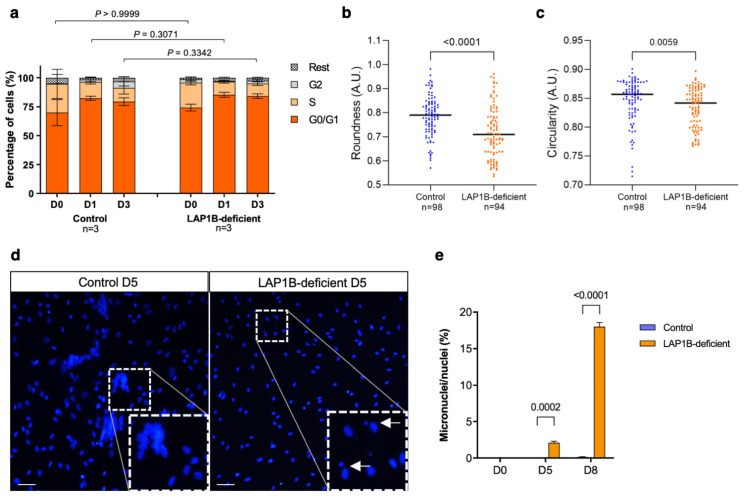
Cell cycle assay and nuclear morphologic and morphometric analyses. (**a**) Percentage of the control and LAP1B-deficient cells in the G0/G1, S, and G2 phases of the cell cycle, as assessed using propidium iodide staining and flow cytometry. *n* = 3, and *p*-values were determined using a two-way ANOVA followed by Bonferroni post hoc tests. (**b**) Roundness and (**c**) circularity of nuclei (A.U.; arbitrary unit) in the control and LAP1B-deficient immortalized fibroblasts. p-values were determined using the Mann–Whitney U test, and n represents the total number of nuclei evaluated in three independent experiments. (**d**) Micrographs of the control and LAP1B-deficient cells at day 5 of differentiation, which were stained using DAPI for nuclei. Arrows indicate the nuclei associated with the micronuclei. Scale bar: 20 μm. (**e**) Percentage of micronuclei relative to the total number of nuclei in the control and LAP1B-deficient cells at days 0, 5, and 8 of differentiation. *n* = 3, and *p*-values were determined using a two-way ANOVA followed by Bonferroni post hoc tests.

Upon induction of myogenic differentiation, proliferating myoblasts must permanently withdraw from the cell cycle and concomitantly activate the transcription of muscle-specific genes [28]. Cell cycle exit depends on MyoD and myogenin expressions, and involves activation of the cyclin-dependent kinase inhibitor p21 and downregulation of E2F target genes [42]. LAP1 localizes in the mitotic spindle during mitotic nuclear envelope breakdown, and both overexpression and knockdown of LAP1 in several cell lines result in post-mitotic defects [13,14,15]. As the LAP1B-deficient cells had an impaired activation of *MYOG*, and LAP1 is involved in cell cycle progression, we investigated proliferation and cell cycle exit in the LAP1B-deficient cells. The control and mutant cells were stained with propidium iodide at days 0, 1, and 3 after MyoD induction, and the cell cycle phases were analyzed using flow cytometry (Figure 6a). At day 0, no significant difference was found in the proportion of cells in the G0/G1 phase, nor in the proportion of cells in the S phase between the controls and the LAP1B-deficient cells (Figure 6a). After induction of differentiation, the percentage of cells in the G0/G1 phase similarly increased in both the control and LAP1B-deficient cells, with no significant difference between the two groups at day 1 and day 3 (Figure 6a). As a result, we found that the LAP1B-deficient cells efficiently exited the cell cycle 24 h after MyoD expression despite delayed *MYOG* expression.

### 2.6. LAP1B-Deficient Fibroblasts Exhibit Nuclear Morphological Defects

Nuclear deformations are a hallmark of nuclear envelopathies and have also been reported in LAP1-associated disorders [17,22,23]. We previously reported ultrastructural alterations of the NE, such as nuclear fragmentation, formation of nuclear blebs and intranuclear canalicules, and chromatin condensation in a muscle biopsy of the patient used in this study [17]. Here, we analyzed the overall nuclear morphology of the LAP1B-deficient fibroblasts before and after MyoD induction. No overt nuclear deformity or micronucleus was detected in the primary and immortalized proliferating fibroblasts before differentiation. However, the quantification of nuclear circularity and roundness in the patient-derived immortalized fibroblasts showed significant decreases in both parameters compared to the controls (Figure 6b,c). Additionally, only after the induction of differentiation that we observed the emergence of micronuclei in the LAP1B-deficient cells (Figure 6d). Indeed, in contrast to the controls, micronuclei were detectable in the LAP1B-deficient cells at day 5 after MyoD induction, and a pronounced increase was observed at day 8 (Figure 6e). These results indicated subtle alterations of the nuclear structures in the patient-derived LAP1B-deficient fibroblasts.

## 3. Discussion

In this study, we established an in vitro model of LAP1B-associated muscular dystrophy by using MyoD-induced myoconversion of patient-derived fibroblasts in order to gain new insights into the molecular mechanisms underlying the pathology. We showed that, despite comparable MyoD expression at both transcriptional and translational levels between the control and mutant cells, the LAP1B-deficient fibroblasts failed to properly initiate and conduct the transcriptional reprogramming towards myogenic differentiation. The mutant cells efficiently exited the cell cycle after MyoD induction; however, they demonstrated strongly decreased differentiation and fusion potentials and, ultimately, died after prolonged exposure to the differentiation cues. Similarly, reduced fusion and differentiation potentials were observed in the LAP1A/B-mutant C2C12 murine myoblasts. These results support the notion that loss of LAP1A/B is sufficient to impair in vitro myogenesis.

We attempted to rescue the myogenic differentiation defect by transiently overexpressing LAP1B in the patient-derived fibroblasts (Figure S3a,b). However, we observed a high mortality (>70%) in the LAP1B-transfected patient’s cells within 24 h and a lack of proliferation, resulting in a failure to maintain cells in the confluent culture for subsequent differentiation (Figure S3a). Moreover, overexpression of hLAP1B in the *Tor1aip1^cl2^* C2C12 myoblasts resulted in a slight but significant increase in the differentiation index compared to the control, but it failed to rescue the fusion defect (Figure S3c,d). A previous study demonstrated that transient overexpression of LAP1B-GFP in HeLa cells causes severe NE aberrations within 48 h and post-mitotic chromosome segregation errors due to the disruption of the correct stoichiometry between LAP1B and torsinA [15]. Therefore, it is possible that the toxicity observed in the LAP1B-transfected patient’s fibroblasts might be a consequence of the disrupted gene dosage. Further experiments should be performed to test whether LAP1B rescue efficiency varies depending on the cell type and the transfection approach (stable vs. transient transfection, transduction vs. transfection, and constitutive vs. inducible expression).

Transcriptome profiling revealed a substantial number of genes differentially expressed in the LAP1B-deficient cells compared to the controls. Overall, the number of downregulated genes in the mutant cells was higher than the number of upregulated genes at each time point, suggesting a failure of transcriptional activation rather than a loss of repression. This is in contrast to the transcriptomic alterations observed in emerin-null myogenic progenitors, where an enrichment of upregulated genes is observed compared to the wild type [43] and is consistent with the loss of repressive transcriptional domains associated with the NE. Further studies are needed to confirm the chromatin state of LAP1B-deficient cells under cycling and differentiation conditions, and to understand the role of the interaction of LAP1B with chromatin.

Several INM proteins, such as LEM (LAP2-emerin-MAN1)-domain proteins and lamin B receptor, are known to directly associate with chromatin [44,45,46,47]. To date, studies on the interaction of LAP1 with chromatin have mainly focused on its role in mitosis, since both knockdown and overexpression of LAP1 are associated with mitotic defects [13,14,15]. Indeed, LAP1 attaches mitotic chromatin to the nuclear envelope during mitosis [15]. LAP1B has been shown to have a higher affinity to mitotic chromatin compared to LAP1C, owing to its longer nucleoplasmic domain [15]. The authors suggested that a low expression of LAP1B in highly proliferative cells may have been favored to reduce the risk of mitotic catastrophes [15]. Hence, it would be interesting to investigate the chromatin interactions of LAP1B in the context of post-mitotic cells—such as during the process of myogenic differentiation—where its expression is increased compared to LAP1C.

In this study, we did not observe proliferation and cell cycle defects in the patient-derived cells (Figure 6a). Similarly, *Lap1*-null primary mouse myoblasts display normal proliferation rates but defective in vitro myogenic differentiation [25]. However, we observed the formation of micronuclei in the LAP1B-deficient cells at day 5 after MyoD induction, as well as altered nuclear morphometric parameters (Figure 6b-e). Emergence of micronuclei is a sign of genotoxic stress, and a previous study demonstrated that LAP1 is implicated in DNA damage response upon genotoxic stress [16]. Interestingly, it has been shown that, upon genotoxic stress, p53 delays myogenic differentiation by inhibiting *MYOG* transcription and inducing p21-mediated cell cycle arrest until the repair of damaged DNA [48]. Therefore, it might be interesting to further investigate the genotoxic stress level in LAP1B-deficient cells, and the molecular pathways underlying the emergence of micronuclei only after the induction of myogenic differentiation.

Transcriptome analyses revealed an initial activation of some muscle genes, such as *TTN* (titin), *TNNT1* (troponin T1), *MYL4* (myosin light chain 4), and *RYR1* (ryanodine receptor 1), upon stimulation of differentiation in the LAP1B-deficient cells and the controls at comparable levels (Figure 4d and Figure S4). However, the LAP1B-deficient cells exhibited a markedly delayed induction of *MYOG* during the first 48 h that remained downregulated throughout differentiation compared to the controls (Figure 5d,f). Similarly, a reduction in the number of myogenin-positive somites was observed in *Lap1*-null mouse embryos [24]. Primary myoblasts isolated from *Lap1*-null embryos displayed defective in vitro myogenic differentiation, such as shorter and thinner myotubes; reduced fusion index; and downregulation of MyoD, myogenin, and MyHC compared to wild type [24]. In MyoD-converted EDMD patient-derived fibroblasts, *MYOG* was shown to be retained at the nuclear periphery [4]. Moreover, human skeletal myogenic cells overexpressing EDMD-associated *LMNA* constructs showed reduced myogenin levels in differentiating myotubes [4]. Therefore, delayed activation of *MYOG* might be a common feature of muscular dystrophies associated with NE proteins and might be caused by altered epigenetic regulation. Further experiments, such as chromatin immunoprecipitation, will help to understand the mechanism of action of LAP1B on the transcriptional regulation of *MYOG.*

In the patient-derived cells, we observed a major fusion defect that might be associated with the downregulation of one of the two major fusogenic genes, *MYMK* (Figure 5c,h). The other fusogenic gene, *MYMX,* was efficiently upregulated in the mutant cells upon differentiation (Figure 5g). Both genes are bound by MyoD and myogenin; however, our results support a previous study suggesting that the activation of *MYMK,* but not *MYMX,* is mainly dependent on myogenin rather than MyoD [41]. Early embryonic depletion of *Lap1* in mouse skeletal muscle causes early postnatal myofiber hypotrophy with reduced myofiber size and overall muscle size without atrophy, while skeletal muscle-conditional knockout of *Lap1* at a later stage results in muscular atrophy [24,25]. Considering that LAP1 is not essential for embryonic muscle development, the authors suggested that the observed muscle hypotrophy could be due to fusion defects of committed myoblasts into mature myotubes. Indeed, they observed impairment in the myogenic potentials of satellite cells (muscle stem cells), with reduced Pax7, MyoD, Myf5, and Mef2C in *Lap1*-depleted mice skeletal muscle [24]. Together, this suggests that LAP1B might have a role in the regulation of factors involved in myogenic fusion.

Interestingly, upon MyoD overexpression, we observed increased protein ubiquitination and decreased ribosomal activity in the LAP1B-deficient cells (Figure 4d). In addition, genes associated with protein digestion and absorption and mTOR signaling were among the most divergently expressed genes throughout differentiation compared to the controls (Figure 5i). Similarly, decreased AKT activity and increased FoxO-mediated atrogene expression were observed in skeletal muscle conditional *Lap1*-null mouse [24]. Altogether, these findings indicate an impaired protein metabolism due to LAP1B deficiency in myogenic cells and support the hypothesis that LAP1 might be involved in the repression of catabolic pathways in muscle.

In conclusion, our results strengthen the hypothesis that LAP1B is critical in muscle cells and its absence is not compensated by LAP1C for efficient myogenic differentiation. This study provides a useful resource for further investigation of the role of LAP1B during in vitro myogenic differentiation using patient-derived cells, and may provide insights into the role of LAP1B in skeletal muscle and NE-related muscular dystrophies.

## 4. Materials and Methods

### 4.1. Immortalized MyoD-Converted Human Fibroblasts

The adult human primary dermal fibroblasts used as the controls were obtained from the American Type Culture Collection (PCS-201-012, Lot no. 64154595 and 63480420). The LAP1B-deficient fibroblasts carrying biallelic c.186delG mutation in *TOR1AIP1* were obtained from a 36-year-old patient affected by LAP1B-related myopathy with rigid spine and distal joint contractures (LGMD2Y, OMIM phenotype number 617072), as previously described [17]. The patient cells were used after written informed consent, and the study protocol was approved by the Hacettepe University Non-interventional Clinical Research Ethics Board (Decision no. GO 16/35).

The primary fibroblasts at passage 2 were immortalized by transduction with a lentiviral vector encoding the catalytic subunit of human telomerase reverse transcriptase, as previously described (a kind gift from Dr. Vincent Mouly) [32]. In addition, cells were transduced with a lentiviral vector expressing *MYOD1* under the control of a doxycycline-inducible Tet-On promoter, as previously described (a kind gift from Dr. Vincent Mouly) [32]. The proliferating cells were cultured in a proliferative medium consisting of high-glucose (4500 mg/mL) Dulbecco’s MEM (DMEM) with stable L-glutamine, supplemented with 10% fetal bovine serum (FBS), 50 µg/mL of gentamycin, and 2.5 µg/mL of amphotericin B, and maintained in 5% CO_2_ at 37 °C. For the induction of MyoD expression, the growth medium was replaced at 90% confluence by a serum-free differentiation medium made with high glucose DMEM supplemented with 2 µg/mL of doxycycline (Sigma-Aldrich, St. Louis, MO, USA) and 10 µg/mL of insulin (Sigma-Aldrich, St. Louis, MO, USA). Transduction efficiency was defined as the efficiency to induce MyoD expression after 18 h of induction, which was determined by comparing the number of MyoD-positive nuclei to the total number of nuclei. For myogenic differentiation, cells were differentiated for 8 days (and up to 17 days for prolonged differentiation experiments) at 37 °C in 5% CO_2_ and by refreshing the differentiation medium every two days.

### 4.2. Tor1aip1-Mutant C2C12 Myoblasts

The C2C12 murine myoblast cell line was obtained from the American Type Culture Collection. Two C2C12 lines harboring loss-of-function mutations in *Tor1aip1* were created using CRISPR/Cas9 tools. The first exon of the mouse *Tor1aip1* gene (Gene ID: ENSMUST00000027738.13) was targeted by two guide RNAs showing minimal off-target sites using the CRISPRDirect software [49]. Oligonucleotides encoding gRNAs (5′-CACCGACCCGTCGCGCCGCGGACGA-3′ and 5′-CACCGTGTACGGCGACTTCGAGCCC-3′) were cloned in pSpCas9(BB)-2A-Puro (PX459) V2.0 (a gift from Feng Zhang; Addgene plasmid # 62988) [50]. After transfection and positive selection with puromycin, single-cell clones of the C2C12 myoblasts were genotyped using Sanger sequencing (forward primer: 5′-AGGTTGGGCCATCTACGTCA-3′, and reverse primer: 5′-GGTCGAGAGCGAAGGTTGTAA-3′), and two *Tor1aip1*-mutant clones were expanded for further analyses. The proliferating C2C12 myoblasts were cultured in high-glucose DMEM with L-glutamine supplemented with 10% FBS, 50 µg/mL of gentamycin, and 2.5 µg/mL of amphotericin B, and maintained in 5% CO_2_ at 37 °C. For myogenic differentiation, the proliferative medium was replaced at >90% confluence by a differentiation medium consisting of DMEM supplemented with 5% horse serum. For the quantification of myogenic differentiation, the mutant clones were compared to the parental wild-type clones at day 5 of differentiation.

### 4.3. Immunofluorescence Staining

The cells were fixed in 4% paraformaldehyde (for MyoD staining) or absolute ethanol (for MF20 staining) and blocked with 1% FBS in a 1X PBS containing 0.1% Triton X-100 for 1 h at room temperature. Primary antibodies were diluted in the 1X PBS to label MyoD (MyoD1, 5.8A, mouse IgG1, Cat. No. MA1-41017, Invitrogen, diluted 1:50) or myosin heavy chains isoforms (MF20, mouse IgG2b, Developmental Studies Hybridoma Bank, Iowa City, IA, diluted 1:20), and incubated for two hours at room temperature. After three washes with the 1X PBS, the cells were incubated for 1 h at room temperature with goat anti-mouse IgG (H+L) Alexa Fluor 488 conjugated-secondary antibody (Invitrogen, Waltham, MA, USA) diluted 1:1000 in the 1X PBS. The cells were washed three times with the 1X PBS, counterstained with DAPI for 5 min, and mounted in a Prolong Gold antifade reagent (Molecular Probes, Eugene, ORE, USA). Micrographs were taken using an Axio Plan microscope (Carl Zeiss AG, Oberkochen, Germany) with an attached AxioCam Erc 5 Mp camera and ZEN 2 software.

### 4.4. Quantification of Myogenic Differentiation

Fusion index and differentiation index were determined at day 5 and day 10 of differentiation to quantify the extent of myogenic differentiation [51]. The cells were differentiated in three independent experiments and stained with MF20 antibody and DAPI at day 0, day 5, and day 10 after MyoD induction. The differentiation index represents the number of nuclei in myosin heavy chain (MyHC)-expressing cells divided by the total number of nuclei. The fusion index represents the number of nuclei in MyHC-expressing cells with at least two nuclei divided by the total number of nuclei. The nuclei from 10 different fields were counted for each sample using ImageJ (Version 1.49u).

### 4.5. RNA Sequencing

The ontrol and LAP1B-deficient immortalized MyoD-converted fibroblasts were seeded as triplicates on three independent culture plates, and differentiation was induced as described above. Total RNA was extracted from one million cells at day 0, day 2, day 5, and day 8 after induction of differentiation using the Hybrid-R total RNA isolation kit (GeneAll Biotechnology, Seoul, Korea), according to the manufacturer’s instructions. Total mRNA libraries for 36 samples were constructed using the SENSE mRNA-seq Library Prep Kit for Ion Torrent (Lexogen GmbH, Vienna, Austria), and sequencing was performed using the Ion S5 System (Ion Torrent, Thermo Fisher Scientific, Waltham, MA, USA) by the Hacettepe University Advanced Technologies Application and Research Center. Quality control of the raw data was performed using FastQC (V0.11.5), and adaptor sequences were removed and reads were trimmed using Cutadapt (V1.18) [52,53]. Reads were mapped to the human reference transcriptome GRCh38 using Salmon (V0.8.1), compensating for GC and sequence-specific biases, and gene abundance was calculated from the read counts per transcript using the tximport package [54,55], based on Ensembl release 86 [56]. After normalization and comparison with a distance matrix, three outliers (samples 6, 21, and 32) were removed. Differential expression was calculated using DESeq2 (V1.20) [57]. The two control samples were averaged and combined as a single control group, and compared to the LAP1B-deficient sample group. For statistical significance, the likelihood-ratio test was applied with the Benjamini–Hochberg False Discovery Rate for multiple comparison correction where q-values represent corrected *p*-values. Genes were considered to be significantly differentially expressed when the q-value was <0.05 and the linear expression fold change was ≥2. A generalized linear model was used as the regression model for the time-course analysis of differentially expressed genes (DEGs). In time-course analysis, expression of genes at each day was compared to day 0, allowing the comparison of genes that were consistently downregulated or upregulated throughout differentiation between the controls and the mutant cells. For the regression analysis, genes were considered to be significantly differentially expressed when the *p*-value was <0.05 and the linear expression fold change was ≥4.

### 4.6. Pathway Analysis

For the comparison of DEGs at each time point between the control and LAP1B-deficient cells, as well as for the comparison of consecutive time points within each group (control or LAP1B-deficient), we used gene set enrichment analysis by submitting gene identifiers and associated log_2_ (fold change) values of significantly DEGs to GeneTrail 3.2 (https://genetrail.bioinf.uni-sb.de/, accessed on 1 August 2022). The Kolmogorov–Smirnov test was used to assess which Gene Ontology (GO)-Biological Process and Kyoto Encyclopedia of Genes and Genomes (KEGG) annotation categories were significantly enriched or depleted in the datasets [58]. The DEGs identified in the time-course regression analysis were analyzed using Enrichr (https://amp.pharm.mssm.edu/Enrichr/, accessed on 1 August 2022) by submitting gene identifiers with a *p*-value <0.05 and an expression fold change of at least 4 (increased or decreased). The top three most significantly enriched (*p* < 0.05) categories of GO-Biological Process and KEGG Pathways were identified [59].

Upstream regulator analyses were generated using Ingenuity Pathway Analysis (IPA) (QIAGEN, Venlo, The Netherlands) (https://www.qiagenbioinformatics.com/products/ingenuity-pathway-analysis, accessed on 1 August 2021) [37]. An upstream regulator analysis examines the number of known targets of a transcription regulator that are present in the dataset and infers if the observed expression change is consistent with significant (*p* < 0.05) transcriptional activation (z-score > 2) or inhibition (z-score < −2) of potential upstream regulators. Transcription regulators present in the dataset with an expression fold change of at least 2 were selected.

### 4.7. Quantitative Real-Time PCR

Total RNA was extracted, as described above, and cDNA was synthesized from 500 nanograms of the total RNA from each sample using the Quantitect Reverse Transcription Kit (QIAGEN, St. Louis, MO, USA), according to the manufacturer’s instructions. Quantitative real-time PCR was performed using the SensiFAST SYBR No-ROX Kit (Meridian Bioscience, Cincinnati, OH, USA) on Rotor-Gene 6000 instrument (Corbett Life Science Pty Ltd., Sydney, Australia). The primer sets are listed in Table S1. The experiments were performed using three biological replicates and two technical replicates. Mean Ct values were obtained using the RotorGene 6000 Series Software 1.8 (Corbett Life Sciences Pty Ltd., Sydney, Australia). Relative expression was calculated using the ΔΔCt method by normalizing the Ct values of *MYOG*, *CDKN1A*, *PTGS2,* and *ANKRD1* to *GAPDH*. Error bars represent mean ± SEM in the figures.

### 4.8. Protein Extraction and Immunoblotting

For protein extraction, cells at corresponding days of differentiation were washed with a cold 1X PBS and collected in a RIPA buffer (Sigma-Aldrich, St. Louis, MO, USA) supplemented with a 1X protease inhibitor cocktail (Roche, Basel, Switzerland) and 1 mM of sodium orthovanadate. The samples were sonicated 6 times at 50% amplitude for 20 s on ice using a Vibra-Cell probe sonicator, VCX 130, probe CV 18 (Sonics & Materials, Newton, CT, USA). After discarding cell debris with centrifugation, protein extracts were quantified using the Bicinchoninic Acid Protein Assay Kit (Thermo Fisher Scientific, Waltham, MA, USA), according to the manufacturer’s protocol, and absorbance at 562 nm was measured using the SpectraMax M2 Microplate Reader (Molecular Devices, San Jose, CA, USA). For SDS-PAGE, 30 µg of protein extracts were denaturated at 100 ºC for 4 min in a 1X Laemmli buffer (Bio-Rad, Hercules, CA, USA). Electrophoresis was performed for 3 h at 100 V, followed by wet transfer to a nitrocellulose membrane (0.45 µM pore size) at 120 V for 90 min. The membranes were blocked with 5% nonfat dried milk in 0.1% Tween 20 in TBS (TBS-T) for 1 h at room temperature. Primary antibodies were diluted in 0.1% TBS-T (1:500 for anti-MyoD1, 5.8A, mouse IgG1, Cat. No. MA1-41017, Invitrogen; 1:2000 for anti-TOR1AIP1 used for human LAP1 detection, rabbit polyclonal, Cat. No. HPA047151, Sigma-Aldrich; 1:2500 for anti-LAP1 used for mouse LAP1 detection, rabbit polyclonal—a kind gift from William T. Dauer; 1:200 for anti-myogenin, clone F5D, mouse monoclonal, Cat. No. sc-1273, Santa Cruz; and 1:5000 for anti-GAPDH, mouse monoclonal, Cat. No. G8795, Sigma-Aldrich), and incubated overnight at 4 °C. After washes, the membranes were incubated with HRP-conjugated secondary antibodies (Invitrogen, Waltham, MA, USA) diluted 1:3000 in 0.1% TBS-T for 1 h at room temperature and stained with a SuperSignal West Femto Maximum Sensitivity Substrate (Thermo Fisher Scientific, Waltham, MA, USA). Chemiluminescence imaging was performed using the GeneGnome 5 instrument (Syngene, Bangalore, India). Full blot images are available in Figure S5.

### 4.9. Cell Cycle Assay

The cells at days 0, 1, and 3 of differentiation that were seeded on three independent plates were washed twice with a 1X PBS and harvested by trypsinization. After centrifugation and washing with the 1X PBS, the cell pellets were fixed with cold absolute ethanol. Propidium iodide staining was performed using the Muse Cell Cycle Reagent (Luminex Corporation, Austin, TX, USA), according to the manufacturer’s protocol. The results were obtained using the NovoCyte flow cytometer (Agilent Technologies, Santa Clara, CA, USA) from at least 10,000 events per sample, and the NovoExpress software (Agilent Technologies, Santa Clara, CA, USA) was used for gating and analyses. Primary gate (P1) was based on forward scatter height (FSC-H) against side scatter height (SSC-H); secondary gate (P2) was based on FSC-H against FCS-area; and tertiary gate (P3) was based on PE-area against width. The cells were selected by excluding cell debris.

### 4.10. Assessment of Nuclear Morphologic and Morphometric Parameters

The cells at day 0, day 5, and day 8 of differentiation were stained with DAPI. The number of micronuclei relative to the total number of nuclei was manually counted using the ImageJ/Fiji software [60]. At least 100 nuclei from three independent plates were counted for each group and time point. Nuclear circularity (4pi∗(area/perimeter^2^)) and roundness (4∗*area/(π∗major_axis^2^)) analyses were performed automatically using the ImageJ/Fiji software [60].

### 4.11. Statistics

The data were statistically analyzed, and graphics were created using GraphPad Prism v.8 (GraphPad Software Inc., San Diego, CA, USA). The results were considered significant when *p* < 0.05. Nonparametric Mann–Whitney U test (two-tailed, unpaired) was used when two groups were compared. One-way ANOVA followed by Tukey’s post hoc tests was used when three groups were compared. Two-way ANOVA followed by Bonferroni post hoc tests was used when three groups at different time points were compared. All error bars are presented as mean ± SEM and bars are represented as median in the figures.

## Data Availability

The data presented in this study have been deposited in NCBI’s Gene Expression Omnibus [61] and are openly accessible through the GEO Series accession number GSE214495 (https://www.ncbi.nlm.nih.gov/geo/query/acc.cgi?acc=GSE214495, accessed on 2 November 2022).

## Supplementary Materials

The following supporting information can be downloaded at https://www.mdpi.com/article/10.3390/ijms232113615/s1.
