# Supplementary material for: Loss of the Nuclear Envelope Protein LAP1B Disrupts the Myogenic Differentiation of Patient-Derived Fibroblasts
Source: Int J Mol Sci. 2022 Nov 6;23(21):13615. doi: 10.3390/ijms232113615 (PMC9656778; doi:10.3390/ijms232113615)
Supplement: Supplementary file 1 [file ijms-23-13615-s001.zip › ijms-1979789-supplementary/Supplementary Figures-revised.pdf]

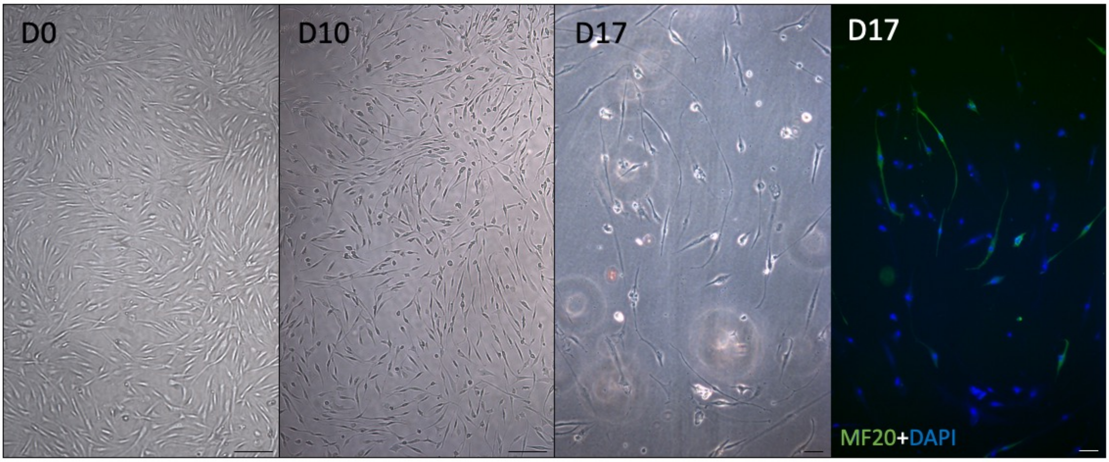

Figure S1. Long-term differentiation of LAP1B-deficient fibroblasts. Phase contrast images of LAP1B-deficient fibroblasts at day 0 (D0) and day 10 (D10) after culture in Dox-supplemented differentiation medium. Cells were fixed and stained at day 17 (D17) with MF20 for myosin heavy chain and DAPI for nuclei. Scale bar: 20 µm.

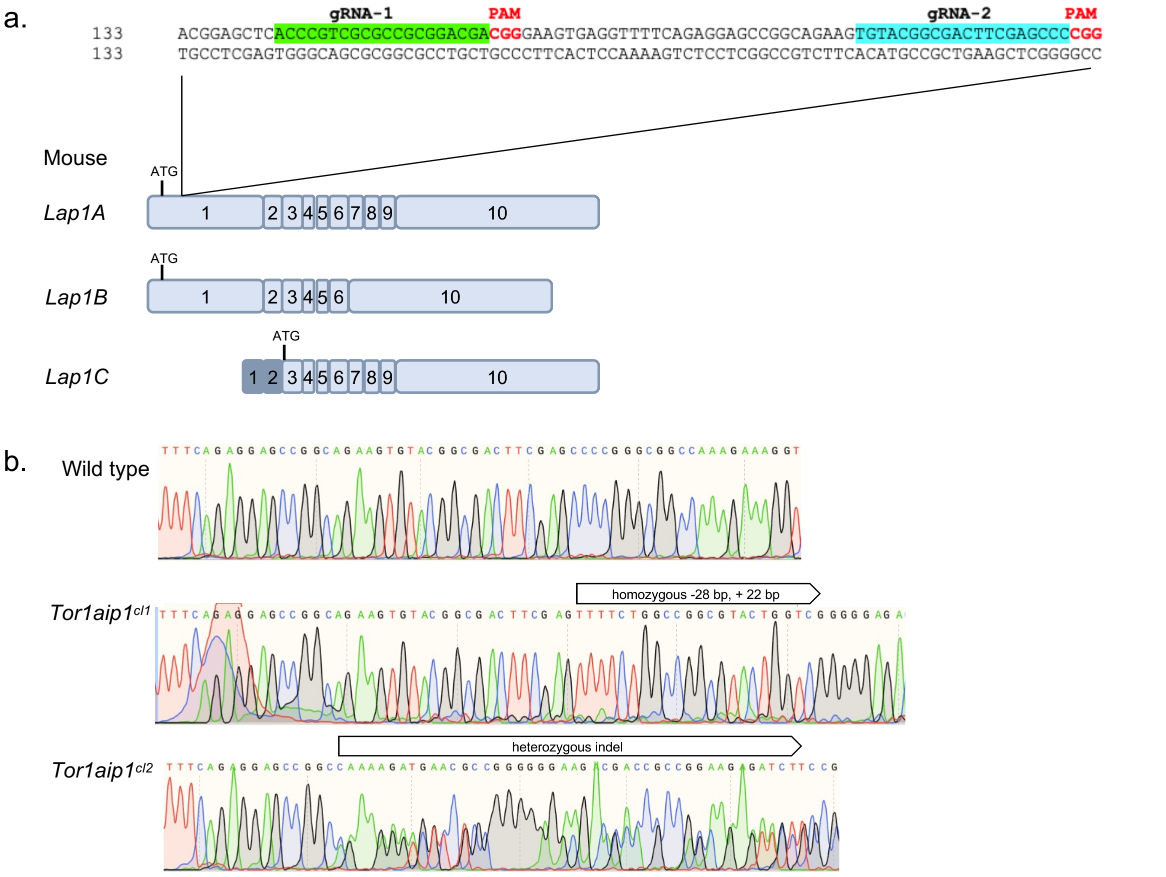

Figure S2. Generation of *Tor1aip1*-mutant C2C12 myoblast lines. (**a**) Schematic representation of mouse *Lap1* transcripts. Each box is numbered according to the corresponding exon in mouse *Tor1aip1*. Position of the start codon is indicated for each transcript. In *Lap1C* transcript, grey boxes represent exonic sequences that are transcribed as UTRs. Positions of gRNAs targeting exon 1 of *Lap1A* and *Lap1B* are highlighted in green and turquoise, with PAM sequence in red. (**b**) Chromatograms of showing mutations in exon 1 of *Tor1aip1* in mutant clone 1 (*Tor1aip1*^{cl1}) and clone 2 (*Tor1aip1*^{cl2}). Wild type sequence is shown in upper panel.

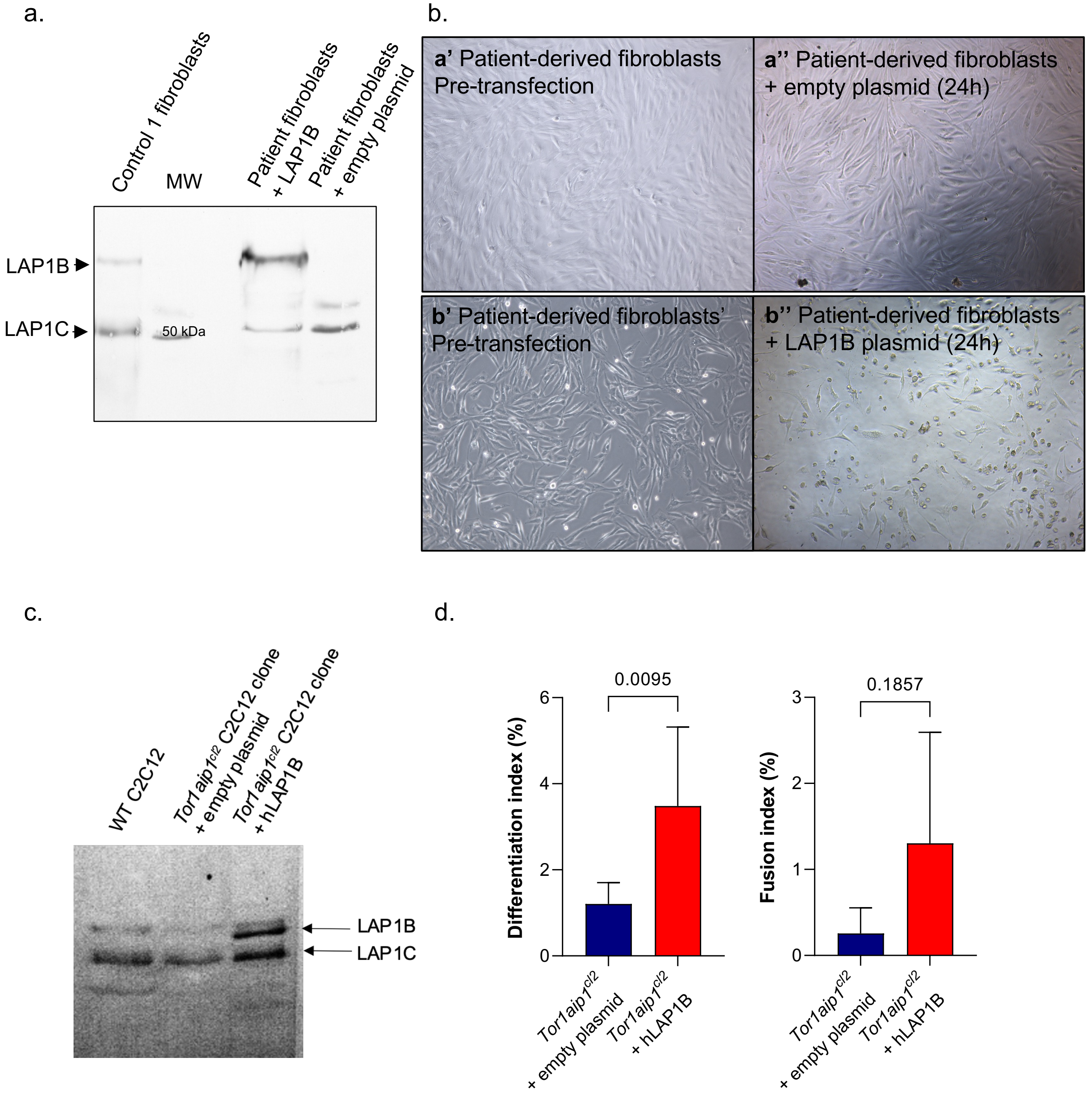

Figure S3. Rescue experiments. (a) Protein expression of LAP1 isoforms in control fibroblasts, patient-derived fibroblasts transfected with pcDNA3.1-hLAP1B, and patient-derived fibroblasts transfected with the empty vector. Membrane incubated with anti-TOR1AIP1 antibody (1:2000, Cat. No. HPA047151, Sigma-Aldrich). MW, molecular weight marker. (**b**) Brightfield images of patient-derived fibroblasts before (a') and 24 h after transfection (a'') of the empty plasmid, as well as before (b') and 24 h after transfection (b'') of pcDNA3.1-hLAP1B. (**c**) Protein expression of LAP1 isoforms in WT C2C12 myoblasts, *Tor1aip1*^{cl2} mutant clone transfected with the empty plasmid, and *Tor1aip1*^{cl2} mutant clone transfected with pcDNA3.1-hLAP1B. Membrane incubated with anti-LAP1 antibody (1:2500, a kind gift from Dr. William T. Dauer). (**d**) Differentiation and fusion indices at day 5 of *Tor1aip1*^{cl2} clone transfected with the empty plasmid, and *Tor1aip1*^{cl2} clone transfected with pcDNA3.1-hLAP1B. n=5, *P* values determined by Mann-Whitney U test.

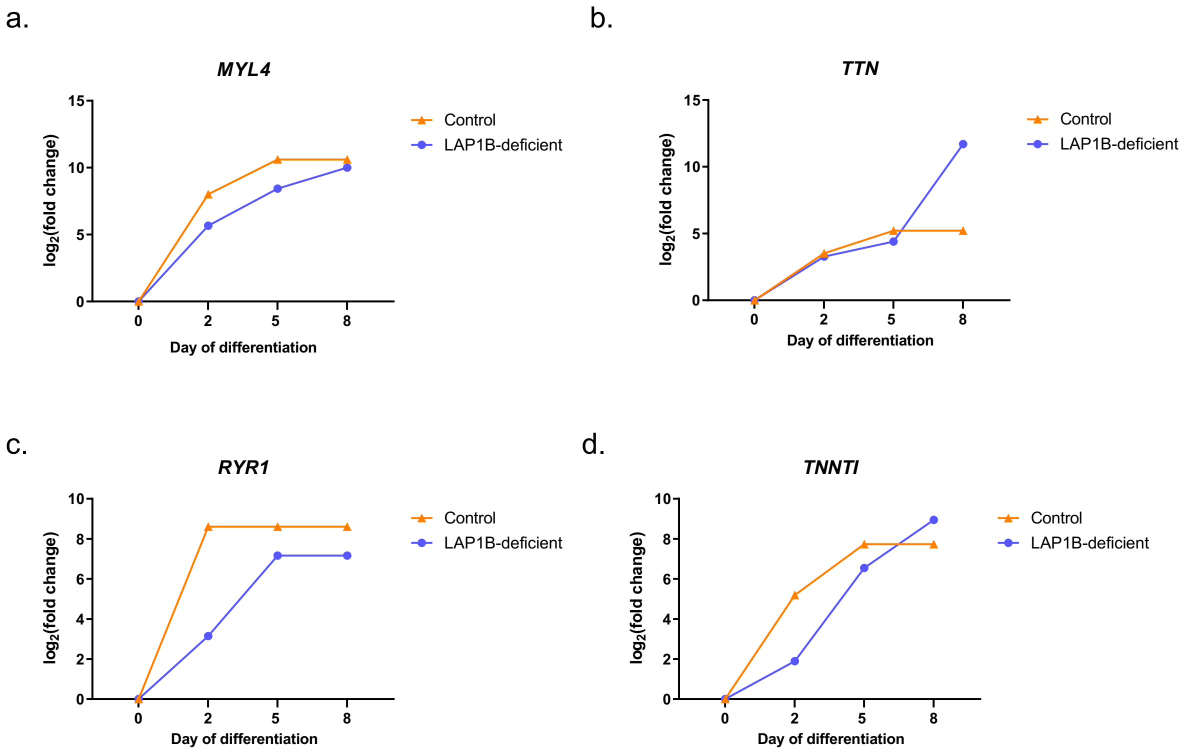

Figure S4. RNA expression pattern of additional muscle-specific genes by RNA-Seq. RNA expression fold changes of (**a**) *MYL4*, (**f**) *TTN*, (**g**) *RYR1* and (**h**) *TNNTI* throughout differentiation determined by RNA-Seq.

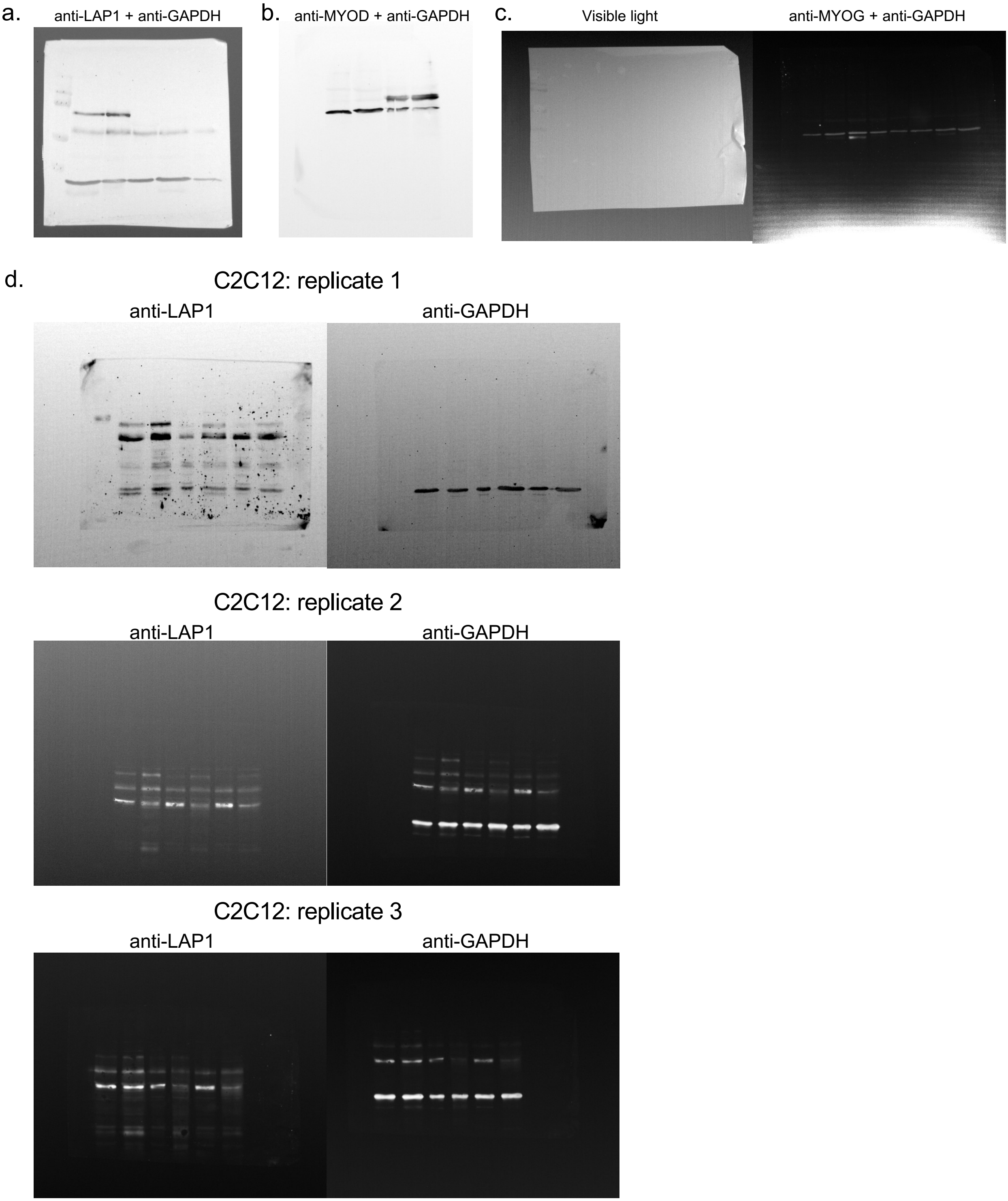

Figure S5. Uncropped blot pictures. (**a**) Membrane in Figure 1a, incubated with anti-TOR1AIP1 antibody (1:2000, Cat. No. HPA047151, Sigma-Aldrich) and anti-GAPDH antibody (1:5000, Cat. No. G8795, Sigma-Aldrich). (**b**) Membrane in Figure 1d, incubated with anti-MyoD1 antibody (1:500, Cat. No. MA1-41017, Invitrogen) and anti-GAPDH antibody (1:5000, Cat. No. G8795, Sigma-Aldrich). (**c**) Membrane in Figure 5d, incubated with anti-myogenin (1:200, Cat. No. sc-1273, Santa Cruz) and anti-GAPDH antibody (1:5000, Cat. No. G8795, Sigma-Aldrich). (**d**) Membranes in Figure 3a, each membrane has been incubated first with anti-LAP1 antibody (1:2500, a kind gift from Dr. William T. Dauer) and photographed, then stripped and incubated with anti-GAPDH antibody (1:5000, Cat. No. G8795, Sigma-Aldrich).

Table S1. Primer sequences used in qPCR.

Gene	Forward primer sequence	Reverse primer sequence
GAPDH	5’GGTCACCAGGGCTGCTTTTA 3	5’TTCCCGTTCTCAGCCTTGAC 3’
CDKN1A	5’AGGTGGACCTGGAGACTCTCAG 3’	5’TCCTCTTGGAGAAGATCAGCCG 3’
PTGS2	5’GTACTCCCGATTGAAGCCCC 3’	5’AAGCCTAATGTGGGGACAGC 3’
ANKRD1	5’GAAAAGCGAGAAACAACGAGA 3’	5’GGTTCCTTTACAACTGGAACTT 3’
MYOG	5’AAACTACCTGCCTGTCCACC 3’	5’ACGGACACCGACTTCCTCTT 3’
